# Genetically Modified Pigs as Organ Donors for Xenotransplantation

**DOI:** 10.1007/s12033-017-0024-9

**Published:** 2017-07-11

**Authors:** Magdalena Hryhorowicz, Joanna Zeyland, Ryszard Słomski, Daniel Lipiński

**Affiliations:** 1 Department of Biochemistry and Biotechnology, Poznań University of Life Sciences, Dojazd 11, 60-632 Poznan, Poland; 2 Institute of Human Genetics, Polish Academy of Sciences, Strzeszyńska 32, 60-101 Poznan, Poland

**Keywords:** Transgenic pigs, Genetic engineering, Xenotransplantation, Genetically modified pigs, Genome modifications

## Abstract

The growing shortage of available organs is a major problem in transplantology. Thus, new and alternative sources of organs need to be found. One promising solution could be xenotransplantation, i.e., the use of animal cells, tissues and organs. The domestic pig is the optimum donor for such transplants. However, xenogeneic transplantation from pigs to humans involves high immune incompatibility and a complex rejection process. The rapid development of genetic engineering techniques enables genome modifications in pigs that reduce the cross-species immune barrier.

## Introduction

With developments in medical science, transplantation of cells, tissues and organs has become a safe and effective method of treating end-stage organ failure or hematopoietic system cancers. The primary problem of transplantology is the disproportion between the number of organs available and the number of patients with indications for transplantation. This disproportion is constantly growing due to the shortage of transplantable organs, combined with the increasing number of indications for this treatment. On average, 18 patients die each day while waiting for a transplant in Europe. Every 10 min, someone is added to the national transplant waiting list. At present, more than 14,500 people are on active organ waiting list (https://www.eurotransplant.org/cms/). The growing shortage of available organs is very difficult to overcome. Currently, two solutions are being considered: extending the life span of patients awaiting transplants and developing new, alternative methods for obtaining transplantable organs. These methods mainly include tissue engineering and the use of stem cells, artificial organs or bioreactors. One especially interesting possibility is xenotransplantation. The use of xenotransplantation in treatment typically involves the transplantation of animal cells, tissues or organs to replace an injured part of the human recipient. At present, the domestic pig (*Sus scrofa domestica*) is considered the best donor of biological material for xenotransplantation. Its anatomical and physiological parameters are similar to humans. The pig breeding is well developed and cost-effective, and the large variety of breeds allows the size of the organs harvested to be matched to the recipient [1]. Unfortunately, the molecular incompatibility between the donor and the recipient, resulting from the large phylogenetic distance between pigs and humans, entails a range of immune complications following transplantation, leading to xenograft rejection. Advances in genetic engineering have made it possible to modify the genome of donor animals in a way preventing the recognition of their organs by the human recipient’s immune system and inhibiting the processes leading to xenograft rejection. Several techniques for obtaining genetically modified animals are available: pronuclear and cytoplasmic microinjection, somatic cell nuclear transfer (SCNT) and viral transduction of DNA. Moreover, transposon-based technologies can be exploited for transgenesis. Both the Sleeping Beauty and the piggyBac systems offer an easy and efficient method for stable genomic insertion. Integration of the exogenous DNA with the host genome may occur randomly—through non-homologous recombination—or through directed transgenesis. Precise genomic modifications are possible with the use of site-specific nucleases, the most recent advancement in biotechnology. These include: zinc finger nucleases (ZFN), transcription activator-like effector (TALE) nucleases and modifications of the CRISPR/Cas (clustered regularly interspaced short palindromic repeats, CRISPR; CRISPR-associated proteins, Cas) bacterial immune system. The first step leading to the introduction of modification in a specific locus in the genome is connected with the induction of double-strand breaks (DSB) in this region. DSB are repaired typically by non-homologous end joining (NHEJ), as a result of which changes may occur in the original nucleotide sequence in DNA or the formation of an indel (insertion/deletion) mutation. If indel mutations appear in the protein-coding sequence, this may lead to the gene frameshift and appearance of premature STOP codons, ending translation. Although transcription progress may be undisturbed, functional protein is not formed. Apart from NHEJ, DSB may be corrected via homologous recombination (HDR). This mechanism may be used to introduce specific changes in a given locus: point mutations, insertion (e.g., transgene insertion) or specific deletions. ZFNs are generated by fusing a DNA-binding domain with the zinc finger motif and a non-specific DNA-cleavage domain comprised of the *Fok*I endonuclease. The *Fok*I domain responsible for DNA hydrolysis requires dimerization of a monomer pair. When two *Fok*I monomers are bound, DNA is cleaved and a DNA double-strand break occurs. Individual zinc finger is responsible for the recognition of three neighboring nucleotides. The DNA-binding domain typically contains between three and six individual zinc finger repeats. In order to generate breaks in the DNA, a ZFN pair is required, in which each DNA-binding domain recognizes a sequence of 9–18 bp. TALENs are similar to ZFNs; however, in this case the zinc finger domains were replaced with DNA-binding domains from proteins of bacteria from the genus *Xanthomonas*. The DNA-binding domain of TALE protein consists of series of 33–35 amino acid repeats that each is responsible for the recognition of a single base pair in DNA. Generally it is difficult, time-consuming and costly to produce proteins recognizing a specific sequence due to the need to produce a separate pair of proteins for each targeted sequence. An alternative to the above-mentioned methods is provided by the CRISPR/Cas system, in which the specificity of DNA recognition is provided not by a protein (as in the case of ZFN or TALEN), but by a short, complementary RNA molecule. CRISPR/Cas9 system is adaptable immune mechanism used by many bacteria to protect themselves from foreign nucleic acids, such as viruses or plasmids. An advantage of the CRISPR/Cas9 system is connected with its high specificity and easy construct design, dependent mainly on the 20 base pairs comprising guide RNA. Apart from the above, multiplexing and expression of several synthetic RNA obtained within one experiment makes it possible to reduce costs and duration of the experiment [2]. The fast development of genetic engineering techniques in recent years has made it possible to perform virtually any kind of genetic manipulation in vitro. Therefore, the prospect of producing multitransgenic pigs whose organs would resist rejection after transplantation is becoming increasingly realistic.

### After the Rejection Response

Due to genetic differences between the donor and recipient, transplantation of xenogenic organs triggers immune response in the recipient, leading to complete failure of the transplanted organ. Depending on the mechanism and timing of xenotransplant rejection, the following classification was adopted: hyperacute rejection (HAR), delayed xenograft rejection (DXR), acute cellular rejection (ACR) and chronic rejection (CR).

#### Hyperacute Rejection

Hyperacute rejection develops within several minutes after organ transplantation and inevitably leads to their failure. The primary role in hyperacute rejection is played by endothelial damage and loss of its biological properties. Pathological changes take place in capillaries and arterioles. We can observe intravascular coagulation, abundant infiltrations mainly composed of neutrophils, leading to necrosis of the transplanted organ. The main reason for hyperacute rejection of the xenograft is the presence of natural, preformed (i.e., existing before the transplantation) antibodies in human plasma that recognize the Galα(1,3)Gal antigen on the surface of porcine endothelial cells. The Galα(1,3)Gal epitope is formed by the bonding of galactose to *N*-acetyllactosamine through α-1,3-glycosidic linkage. The catalyst for the reaction is the α-1,3-galactosyltransferase (GGTA1) enzyme. The recognition and binding of the Galα(1,3)Gal antigen by xenoreactive antibodies activates the classical complement pathway, leading to the formation of the membrane attack complex (MAC), which acts as a catalyst for cell membrane penetration by proteins forming transmembrane channels, ultimately resulting in cell lysis [3].

To obtain transplantable organs from the animal donor, the Galα(1,3)Gal antigen must be removed from xenograft cell surfaces. The best method to rid off Galα(1,3)Gal epitopes is the inactivation of the gene encoding GGTA1, which acts as a catalyst for the Galα(1,3)Gal epitope forming reaction. Genetic recombination can be used to replace the wild-type GGTA1 gene with a mutant variant, preventing the production of the enzyme. In 2001, pigs with heterozygously inactivated GGTA1 were produced [4], and 1 year later, piglets with homozygous inactivation of the gene [5]. The first xenotransplantation using hearts from GGTA1-inactivated (GTKO) pigs was performed in 2005. Their recipients were immune-suppressed baboons. The mean graft survival period was 92 days, and the longest surviving graft functioned in the recipient for 179 days [6]. Petersen et al. using intracytoplasmic microinjection of CRISPR/Cas9 vector obtained biallelic knockouts of GGTA1 gene in three of six piglets. This simplified injection method avoids the penetration of the pronuclear membrane, what results in increasing survival rates of microinjected embryos. Moreover, the use of such an efficient technology as CRISPR/Cas9 significantly facilitates and shortens process of generation pigs with multiple genetic modifications for xenotransplantation [7].

Further research is being conducted to identify and eliminate other surface xenoreactive antigens from porcine cells. Pigs with inactivated GGTA1 and the gene encoding cytidine monophosphate-N-acetylneuraminic acid hydroxylase (CMAH), which catalyzes the reaction producing the Neu5Gc antigen (*N*-glycolylneuraminic acid), have been produced. Neu5Gc is not expressed in humans, but is found on cell surfaces in Old World monkeys. The authors of the study showed that cells from these double knockout pigs were better protected against human serum than cells from GTKO animals [8]. Moreover, Butler et al. [9] demonstrated that the inactivation of both the GGTA1 and CMAH genes in pigs significantly reduced the xenogeneic consumption of human platelets in a liver perfusion model.

Another xenoreactive antigen found on porcine cell surfaces, to which humans and non-human primates have antibodies, is a glycan produced by the β1,4-*N*-acetylgalactosaminyltransferase (β4GalNT2) enzyme activity [10]. Estrada et al. used the CRISPR/Cas9 technique to produce pigs lacking the GGTA1, CMAH and β4GalNT2 genes. In vitro tests showed reduced human IgM and IgG binding to PBMCs from genetically modified animals (GGTA1/CMAH/β4GalNT2-inactivated) compared to cells from pigs lacking the Galα(1,3)Gal and Neu5Gc epitopes, which suggests that the elimination of the glycan produced by β1,4-*N*-acetylgalactosaminyltransferase activity may be a useful strategy of genetic modification in pigs used for xenotransplantation [11].

However, xenoreactive antibodies are the main cause for hyperacute rejection; complement system plays a central pathophysiological role in this type rejection. Some factors and mechanisms exist that can be used to regulate complement activity. The most promising ones with regard to xenotransplantation include CD46, CD55 and CD59. CD46, membrane cofactor protein, protects cells expressing it by blocking the formation of the C3 convertase complex, which takes place in the complement activation pathway. CD55, the decay-accelerating factor, regulates cell susceptibility to complement attack. Its role consists in inhibiting the formation of C3 and C5 convertases and accelerating their decay. As to CD59, membrane inhibitor of reactive lysis, it prevents the formation of MAC, which is the final stage of the complement enzyme cascade. CD59 protects cells from lysis by binding to complement C8 and C9, blocking C9 polymerization and cell membrane attack. High expression of human (h)CD59 in pigs has been shown to protect organs from these animals against complement attack when perfused ex vivo with human blood [12]. Transgenic pigs with both the hCD59 and the hα1,2-fucosyltransferase (which reduces the expression of the Galα(1,3)Gal epitope) transgenes were also produced. Aortic endothelial cells (AEC) and peripheral blood mononuclear cells (PBMC) from these animals were more resistant to complement-dependent lysis than cells from monotransgenic animals with either the CD59 transgene or the α1,2-fucosyltransferase transgene [13]. Resistance to complement attack was also induced by modifying the porcine genome with a gene construct containing a CD55 encoding sequence. Kidneys from such transgenic pigs transplanted into macaques having undergone splenectomy and immune suppression using cyclosporin A, cyclophosphamide and steroids remained functional in the recipient for up to 78 days [14]. Hearts from CD55 transgenic pigs transplanted into baboons, with immunosuppression, functioned for up to 139 days [15]. The introduction of the hCD46 gene into the porcine genome resulted in a high expression of the protein, protecting organs from such transgenic pigs against destruction by the recipient’s complement system. Hearts from these transgenic pigs transplanted into baboons, with splenectomy and immunosuppressants (tacrolimus, sirolimus), functioned for up to 137, 96 days on average [16]. In 2012, baboons were transplanted with hearts from GTKO/hCD46 pigs. With added immune suppression and B cell depletion, the maximum xenograft survival time in the recipient was extended up to 236 days [17]. Transgenic pigs expressing all three complement-regulating factors, CD46, CD55 and CD59, have also been produced. Research demonstrated that cells from triple-transgenic pigs were more resistant to complement attack than double-transgenic (CD55 and CD59), monotransgenic or non-transgenic ones [18].

#### Delayed Xenograft Rejection

A xenogenic transplant, which avoids hyperacute rejection, is subjected to delayed xenograft rejection, taking place several hours to several days after organ transplantation [19]. The course of delayed rejection resembles closely hyperacute rejection, with this difference that it does not require the participation of a complement, it involves IgG immunoglobulins, and it develops more slowly and is initiated in the arterial lumen and not in capillaries or arterioles. During delayed xenotransplant rejection endothelial cells undergo type II activation. We observe increased expression of many proinflammatory genes, increased secretion of chemokines and blood platelet activation.

The coagulative disorders are the result of molecular differences between the pig and human coagulation systems. CD39 is an ectoenzyme present in the vascular endothelium and in blood cells, which plays an important role in the regulation of clotting and inflammatory processes. Its activity inhibits platelet aggregation triggered by adenosine triphosphate (ATP) and adenosine diphosphate (ADP) release. CD39 hydrolyzes extracellular ATP and ADP into adenosine monophosphate (AMP) and adenosine, the latter being a strong platelet aggregation inhibitor. ATP is also metabolized into adenosine by the CD73 (ecto-5′-nucleotidase) enzyme. Compared with human cells, porcine endothelial cells have much lower CD73 levels, which causes reduced adenosine production, leading to intravascular coagulation [20]. Therefore, the introduction of hCD39 and hCD73 genes into the porcine genome has been proposed to increase adenosine production. hCD39 expression in pigs has been shown to significantly protect against myocardial damage in an ischemia–reperfusion model [21].

Thrombomodulin (TM) is an integral membrane protein expressed on the surface of endothelial cells, playing a role in coagulation inhibition. Bound to thrombin, TM activates protein C, which is strongly anticoagulative. Protein C is a protease which, with protein S as a cofactor, inhibits factors Va and VIIIa, inactivating the enzymatic cascade causing clot formation. The inability of porcine TM to bind human thrombin prevents protein C activation [22]. Petersen et al. successfully generated transgenic pigs with hTM gene by somatic cell nuclear transfer. They demonstrated that hTM expressing porcine fibroblasts showed elevated activated protein C production in an in vitro TM coactivity assay [23]. Moreover, Miwa et al. [24] showed that hTM expression in porcine AECs inhibits prothrombinase activity and delays coagulation. Mohiuddin et al. [25] using GTKO/hCD46/hTM pigs and high doses of anti-CD40 immunosuppressants achieved the longest xenograft survival time (945 days) so far, in heterotopic pig-to-non-human primate cardiac xenotransplantation model.

The vascular endothelium produces the strongest natural extrinsic coagulation pathway inhibitor—TFPI (tissue factor pathway inhibitor). The anticoagulant activity of TFPI consists in reversible inhibition of factor Xa and formation of the Xa/TFPI complex, which subsequently inhibits the TF (tissue factor)/VIIa complex. TFPI thus plays a double role in the coagulation process, inhibiting both factor Xa and the TF/VIIa complex. It is the only inhibitor acting at the early stage of coagulation, preventing the formation of thrombin. Porcine TFPI is not an effective inhibitor of human factor Xa and may be ineffective in deactivating TF [26]. TFPI overexpression has great potential for controlling the initiation of TF-dependent coagulation in xenotransplantation. Iwase et al. [27] showed that human platelet aggregation induced by porcine AECs with the hTFPI gene was lower than that induced by cells from non-transgenic pigs. Reduction of TF expression could offer another solution. A team headed by Niemann used small interfering RNA (siRNA) to knock down the TF gene, producing transgenic pigs with an expression of TF decreased by 94.1%. The modification extended the coagulation time and decreased clotting compared to non-transgenic pigs used as controls [28].

The von Willebrand factor (vWF) is a glycoprotein playing a key role in coagulation. This factor is necessary for platelet adhesion at the site of the vascular damage. It binds to factor VIII (the protein required for clot formation) in the blood, protecting it from premature decay. The von Willebrand factor plays a significant role in xenograft rejection, especially in the case of xenogeneic lungs, which release more vWF than hearts or kidneys [29]. One solution to this problem is the use of organs from pigs with inactivated vWF gene. Such animals have already been produced using the latest CRISPR/Cas9 technology [30].

Excess accumulation of fibrin can be prevented by an effective protein C anticoagulant system. EPCR increases activated protein C (APC) activation, which has anticoagulant properties, by the thrombin–TM complex [31]. Furthermore, EPCR forms a complex with APC, triggering a response in endothelial cells, which decreases proinflammatory cytokine synthesis. Therefore, the introduction of a hEPCR-encoding gene into the porcine genome has been suggested in order to regulate inflammatory processes and decrease the risk of thrombosis. Using such a modification, Iwase et al. [27] showed a positive correlation between decreased human platelet aggregation and hEPCR expression in porcine AECs. Porcine lungs with overexpressed hEPCR functioned longer than controls and were associated with decreased platelet activation in a xenogeneic reperfusion model [32].

Another approach to inhibiting delayed xenograft rejection is introducing anti-inflammatory and antiapoptotic genes, protecting porcine endothelial cells, into the recipient’s genome. Heme oxygenase 1 (HO-1) is an enzyme participating in the degradation of heme into iron ions (Fe^2+^), carbon monoxide (CO) and biliverdin. Heme degradation products are important biologically active compounds, which contribute to the protection of cells against apoptosis, free radical formation and inflammation [33]. The anti-inflammatory and antiapoptotic properties of HO-1 have also been used in xenotransplantation studies. It has been shown that porcine AECs with the hHO-1 gene are protected against TNF-α-dependent apoptosis. HO-1 overexpression also increased the survival of transgenic animal organs in an ex vivo kidney perfusion model, compared to controls [34].

Zinc finger protein A20, tumor necrosis factor alpha-induced protein 3, acts in an anti-inflammatory manner by inhibiting the activity of the nuclear factor kappa-light-chain-enhancer of B cells (NF-κB) and inhibits TNF-mediated programmed cell death [35]. Human A20 expression in pigs has been shown to have significant immune-modulating effects in porcine AECs, making them less susceptible to cell death induced by Fas (CD59) ligands [36]. Moreover, Fisher et al. produced pigs expressing human HO-1, A20, CD46, CD55 and CD59 genes and confirmed the antiapoptotic effects of A20 and HO-1 in those pigs. After treatment with human TNF-α and cycloheximide, porcine kidney fibroblasts from multitransgenic pigs showed inhibition of caspase 8 induction. The groundbreaking importance of this study was to obtain pigs with multiple xenoprotective transgenes collocate at single genomic locus; therefore, these introduced genes are transmitted to the next generation without any segregation. Furthermore, the transgenes were placed into ROSA26 locus which provides a high and consistent expression of foreign genes without interrupting the function of essential endogenous genes [37].

#### Cellular Rejection

ACR occurs within several days after transplantation. The dominant morphological characteristics of such rejection are infiltrations with predominant mononuclear cells, located interstitially in tissues. In the human the cellular response targeting porcine tissues is induced both by NK cells and by T lymphocytes, with that CD4 + lymphocytes exhibit a much higher level of cytotoxicity than CD8 + lymphocytes [38]. The susceptibility of porcine endothelial cells to lysis induced by human NK cells is to the inability of NK cell inhibitory receptors to identify major histocompatibility complex (MHC) class I molecules, resulting from differences between porcine (swine leukocyte antigens, SLA) and human (human leukocyte antigens, HLA) MHC [39]. The introduction of the HLA-E gene into porcine endothelial cells has been shown to partially protect the porcine endothelial cells in vitro [40]. Miyagawa’s team demonstrated in turn that the introduction of HLA-E into pigs’ genome protects porcine organs not only against NK cytotoxicity, but also against macrophage cytotoxicity [41]. Furthermore, Laird et al. [42] showed that relative to GTKO/hCD46 pig lungs perfused with human blood, additional expression of HLA-E (GTKO/hCD46/HLA-E lungs) increased median lung survival ex vivo and was associated with reduced pulmonary vascular resistance and decreased platelet activation.

Another cause of cytotoxicity leading to lysis of porcine endothelial cells is the binding of porcine UL-16-binding protein 1 (ULBP1) to NKG2D receptors. Lilienfeld et al. have demonstrated that cytotoxicity against porcine endothelial cells mediated by freshly isolated or IL-2-activated NK cells through NKG2D was completely blocked using anti-ULBP1 polyclonal antibodies. This suggests that ULBP1 is the primary functional ligand in porcine cells for the human NKG2D receptor [43].

Macrophages are a functionally heterogeneous population of mononuclear cells, playing an important role in inflammatory processes, as phagocytic cells. They may be activated either by xenoreactive T cells or by a direct interaction between donor endothelial antigens and macrophage surface receptors [44]. Primate macrophages have been shown to participate in the rejection of porcine pancreatic islets with Galα(1,3)Gal removed and to phagocytose porcine red blood cells independently of antibodies or the complement system [45]. Phagocytosis is inhibited by the CD47 surface antigen [46]. CD47, integrin-associated protein, is a member of the Ig superfamily expressed in all tissues. The signal regulatory protein α (SIRPα) present on the cell membrane is an inhibiting receptor recognizing CD47 [47]. The CD47–SIRPα interaction delivers a phagocytosis-inhibiting signal to the macrophages. CD47 present on porcine endothelial cell surfaces differs from its human counterpart. Ide et al. [48] showed that such binding of porcine CD47 by human SIRPα does not have an inhibitory effect on human macrophages. The authors demonstrated that hCD47 expression in porcine cells decreased their susceptibility to macrophage phagocytosis in vitro. Tena et al. [49] produced GTKO/hCD47 pigs and demonstrated that the modification effectively inhibited macrophage activation and phagocytosis of the transgenic animal cells. The same author demonstrated that expression of hCD47 on hematopoietic cells of GTKO pigs substantially increases the transient xenogeneic chimerism in baboons receiving hematopoietic cells, leading to markedly prolonged survival of porcine skin grafts in the absence of concurrent immunosuppression [50]. Moreover, Jung et al. demonstrated that in addition to the hCD47–SIRPα interaction, hTFPI expressed by transgenic porcine cells enhanced the hCD47-SIRPα axis. It might improve the effect of hCD47-SIRPα signaling and help overcome macrophages-mediated immune rejection [51].

#### Chronic Rejection

Chronic rejection occurs within months or even years after transplantation, and it is clinically manifested by progressing failure of the grafted organ or tissue. It is assumed that chronic rejection develops as a result of additive effects of various harmful factors. At present little is known on chronic xenotransplant rejection. It is suggested that the initiating factor may be connected with damage of the vascular endothelium by cytotoxic T lymphocytes and specific antibodies. However, the intensity of the response is much lower than in the case of acute rejection. In cell membranes we observe increased expression of cell adhesion molecules and the tissue factor activating the coagulation system. Gradual proliferation of the aortic media takes place, leading as a consequence to vascular obliteration and organ failure.

### Risk of Viral Infection with Xenotransplantation

The use of porcine cells, tissues or organs for xenotransplantation raises concerns about the risk of infection with viruses present in the animals. Most pathogens that could potentially be harmful to humans can be eliminated by careful donor selection, breeding in sterile and isolated conditions and screening of each donor candidate. Porcine endogenous retroviruses (PERV) remain a problem, as they constitute an integral part of the porcine genome [52]. Endogenous retroviruses are dormant (inactive) in the host, causing no disease symptoms. They may, however, be activated by certain factors and thus become infectious. If an endogenous virus is present in the xenograft, it could potentially become activated and pathogenic in the recipient. Based on nucleotide sequence differences within the *env* gene, encoding viral envelope glycoproteins, porcine endogenous retroviruses can be divided into three subtypes: PERV-A, PERV-B and PERV-C [53]. Research conducted so far indicates that the PERV-A and PERV-B subtypes are present in various pig breeds in all the populations studied, while PERV-C is not found in all animals [54]. Both PERV-A and PERV-B can infect various human cell types in vitro [55]. The situation is different with regard to the PERV-C subtype, which is practically incapable of infecting human cells by itself, but can, through recombination with the PERV-A subtype, exhibit increased infectivity toward human cells compared with the PERV-A subtype alone [56].

PERVs are very difficult to eliminate, as they are encoded in multiple locations in pig genome [57]. To reduce the risk of PERV infection in humans, it has been proposed that xenograft donor candidate animals should be tested for retrovirus levels, so that organs can be harvested only from those with low values, while carriers of the PERV-C subtype should be eliminated altogether. Another suggested solution involves the use of small interfering RNA (siRNA) [58, 59] and other genome editing techniques (ZFN, TALEN and CRISPR/Cas) to remove PERV-encoding sequences from the animals’ genome. For this strategy to succeed, the technique used must deactivate dozens of very similar genes at once. This is why the CRISPR/Cas method is the most promising, as it allows for simultaneous modification of multiple parts of the genome. Using this technology, Yang et al. [60] designed two RNA molecules to inactivate 62 copies of the *pol* gene required for PERV activity. The study on a porcine kidney epithelial cell line demonstrated that the modification produced a 1000-fold reduction in PERV transmission to human cells, compared to non-transgenic control cells, giving rise to great hopes for the complete elimination of these viruses from pigs used as xenograft donors.

## Conclusions

Genetically modified pigs hold great promise in xenotransplantation. Therefore, genetically modified pigs can become cell, tissue and organ donors, providing a solution to severe shortage of organ donors. Advances in genetic engineering have made it possible to modify the xenograft donor genome in virtually unlimited ways. The challenge facing researchers is to develop the most effective combination of donor genome modifications to overcome the multilayered obstacles to xenotransplantation. The development of transplantation medicine would not have been possible without immunosuppressive drugs, which are also used in research on xenograft rejection inhibition. Some most commonly used substances include: mycophenolate mofetil, tacrolimus, sirolimus, cyclosporin, belatacept, abatacept, fingolimod and everolimus [61, 62]. Immunosuppressive drugs should be selective and administered in appropriate doses, so as to suppress the processes related to xenograft rejection on the one hand, while allowing normal immune responses to any infectious process in the recipient on the other. Table 1 summarizes the most important results and the longest survival times in organ pig-to-non-human primates models using genetically modified pigs and immunosuppressive drugs.

**Table 1:** Survival of organs from genetically modified pigs into non-human primates

Type of graft	Type of genetic modification	Immunosuppression	Longest survival time (days)	Year (References)
Heart (heterotopic transplantation)	GTKO, CD46, TM	ATG, CD20, CVF, CD40, MMF, CS	945	2016 [63]
	GTKO, CD46	ATG, CD20, CD154, CVF, MMF, CS	236	2012 [17]
	GTKO	ATG, TI, CD2, CD154, CVF, MMF, MP	179	2005 [6]
Heart (orthotopic transplantation)	CD46	ATG, or CyP, CD20, TAC, Rapa	57	2011 [64]
	GTKO, CD46, TM	CD40, ATG, CD20, MMF; non-ischemic preservation technique	40	2017 [65]
	CD55	CyP, CsA, MMF, CS	39	2000 [66]
Kidney	GTKO, CD55	CD4, CD8, CD154, MMF, CS	310	2016 [67]
	GTKO, CD46, CD55, EPCR, TFPI, CD47	ATG, CD20, CD40, Rapa, CS	237	2017 [68]
	CD55	ATG, MMF, CS, CD2, CD154, TAC, CyP, CVF, thymectomy or thymic irradiation	229	2003 [69]
	GTKO, CD46, CD55, TM, EPCR, CD39	ATG, CD20, CD40, Rapa, MP, CVF,	136	2015 [70]
Liver (orthotopic transplantation)	GTKO	ATG, CVF, CTLA4-Ig, TAC, CS	25	2016 [71]
	CD55	CyP, CsA, CS	8	2000 [72]
Lung (orthotopic transplantation)	vWF-KO	CsA, INN, AZA, CS, macrophage-depleted, antibody immunoabsorption	109 h	2007 [73]
	GTKO, CD46	CS + CsA + AZA, macrophage-depleted	48 h	2011 [74]

In the case of lung xenotransplants, survival is given in hours

*ATG* antithymocyte globulin, *AZA* azathioprine, *CD154* antihuman CD154 (CD40L), *CD2* rat antihuman CD2 (LoCD2b), *CD20* antihuman CD20 (rituximab), *CD4* anti-CD4, *CD40* antihuman CD40, *CD8* anti-CD8, *CS* corticosteroids, *CsA* cyclosporin, *CVF* cobra venom factor, *CyP* cyclophosphamide, *INN* indomethacin, *MMF* mycophenolate mofetil, *MP* methylprednisolone, *Rapa* rapamycin (sirolimus), *TAC* tacrolimus (FK-506)

The concept of xenotransplantation is relatively old, but for many years, any effective applications remained beyond the realm of possibility. Limitations in both knowledge and technology were too great and multifaceted to render this idea true. Xenotransplantation is a multidisciplinary undertaking, requiring the development of a range of research methods. The range of specialties involved is broad, from molecular biology (developing the appropriate gene constructs, determining the characteristics of the transgenic animals produced), to animal breeding and experimental embryology (introducing gene constructs), pig farming (raising the pigs), immunology (ensuring donor and recipient histocompatibility), virology (detecting endogenous retroviruses), to transplant surgery. In recent years, advances have been made in all these areas, in terms of both knowledge and technology, bringing the successful application of xenotransplantation closer to reality.
